# Validation of SYBR green I based closed‐tube loop‐mediated isothermal amplification (LAMP) assay for diagnosis of knowlesi malaria

**DOI:** 10.1186/s12936-021-03707-0

**Published:** 2021-03-25

**Authors:** Meng Yee Lai, Choo Huck Ooi, Yee Ling Lau

**Affiliations:** 1 Department of Parasitology, Faculty of Medicine, University of Malaya, Kuala Lumpur, 50603 Malaysia; 2 Sarawak State Health Department, Jalan Diplomatik, Off Jalan Bako, Kuching, Sarawak 93050 Malaysia

**Keywords:** *Knowlesi*, LAMP, SYBR green I, Malaria, Molecular diagnostic

## Abstract

**Background:**

As an alternative to PCR methods, LAMP is increasingly being used in the field of molecular diagnostics. Under isothermal conditions at 65 °C, the entire procedure takes approximately 30 min to complete. In this study, we establish a sensitive and visualized LAMP method in a closed-tube system for the detection of *Plasmodium knowlesi*.

**Methods:**

A total of 71 malaria microscopy positive blood samples collected in blood spots were obtained from the Sarawak State Health Department. Using *18s rRNA* as the target gene, nested PCR and SYBR green I LAMP assay were performed following the DNA extraction. The colour changes of LAMP end products were observed by naked eyes.

**Results:**

LAMP assay demonstrated a detection limit of 10 copies/µL in comparison with 100 copies/µL nested PCR. Of 71 *P. knowlesi* blood samples collected, LAMP detected 69 microscopy-positive samples. LAMP exhibited higher sensitivity than nested PCR assay. The SYBR green I LAMP assay was 97.1% sensitive (95% CI 90.2–99.7%) and 100% specific (95% CI 83.2–100%). Without opening the cap, incorporation of SYBR green I into the inner cap of the tube enabled the direct visualization of results upon completion of amplification. The positives instantaneously turned green while the negatives remained orange.

**Conclusions:**

These results indicate that SYBR green I LAMP assay is a convenient diagnosis tool for the detection of *P. knowlesi* in remote settings.

**Supplementary Information:**

The online version contains supplementary material available at 10.1186/s12936-021-03707-0.

## Background

Malaria is one of the global health diseases caused by the deadly parasite *Plasmodium* spp. which requires a rapid and reliable diagnosis tool. Goals have been set world-wide to achieve the aim of malaria eradication programmes. In Malaysia, most malaria cases are contributed by *Plasmodium knowlesi* which was previously thought to only infect monkeys. This parasite has now been shown to infect humans as well [1, 2].

Early and accurate malaria diagnosis is necessary to enable effective treatment. Microscopic examination is the gold standard in malaria diagnosis. However, the limitation of microscopy in detection of low parasitaemia samples is of great concern. The results can only be read by experienced and well-trained personnel. Recently, some rapid test kits were made available as a tool for malaria diagnosis. However, malaria cases may be undetected due to low parasitaemia and improper storage conditions of the kits. The results may vary depending on different types of rapid test kits used. Foster et al. [3] reported that patients with *P. knowlesi* were misdiagnosed as *Plasmodium falciparum* by OptiMAL-IT test kit or *Plasmodium vivax* by Paramax-3 test kits. Patients with *P. knowlesi* also could be misdiagnosed as non-*P. vivax/non-P. falciparum* with BinaxNOW® Malaria kit. Misdiagnosis of *Plasmodium* spp. results in inaccurate administration of anti-malarial drugs, leading to increased severity of the disease [3].

In order to improve the sensitivity of the rapid test kits, molecular methods are widely used. Nested PCR is highly sensitive with a low detection limit of 1–5 parasites/µL [4]. However, the long turnaround time and requirement of expensive equipment and PCR reagents render this method inadequate for routine diagnosis in low-resource settings.

Therefore, isothermal nucleic acid amplification methods are an alternative to PCR in malaria diagnosis due to the cheaper equipment required. LAMP-based assays are the most widely reported among the isothermal methods [5]. Unlike PCR, which requires cycling parameters, LAMP is able to amplify nucleic acids at a constant temperature ranging from 60–65 °C. Further innovations of this technology enable LAMP to be developed as a portable format for field use. The amplified products can be visualized via visual judgment of the turbidity or fluorescence of the end products.

In this study, SYBR green I LAMP assay was designed for the visual detection of LAMP products by direct observation of the end products’ colour changes. SYBR green I dye is a green fluorescent cyanine dye that has high affinity for double stranded DNA as well as RNA. During the amplification, SYBR green I dye will bind to nucleic acid molecules and fluorescence increases upon the accumulation of DNA molecules [6].

## Methods

### Sample collection and DNA extraction

A total of 71 malaria positive and 20 healthy blood samples collected in blood spots were obtained from the Sarawak State Health Department for this study. Samples were collected dated from year 2015 to 2020. DNA was extracted from blood spots using DNeasy® Blood and Tissue Kit (Qiagen, Hilden, Germany) according to the manufacturer’s protocol. The samples were examined under a microscope prior to DNA extraction. Parasitaemia of the collected samples ranged from 0.30 to 3.87% (15,000 to 179,000 parasites/µL). Ethics approval was obtained from the Medical Research and Ethics Committee (MREC) of the Ministry of Health Malaysia (NMRR-15-672-23975) and the Medical Ethics Committee of University of Malaya Medical Centre (MEC Ref. No. 817.1). All malaria samples tested by LAMP or nested PCR were collected prior to antimalarial treatment. All malaria samples collected were confirmed by microscopist at the hospital and cross-checked by Medical Laboratory Technician at the District Health Office.

### Nested PCR

Species identification was carried out by nested PCR targeting *Plasmodium* 18 small subunit ribosomal RNA gene (*18S rRNA*). Primers used were synthesized according to Snounou et al. [7] (Table 1). The cycling parameters and conditions have been described in a previous study [8].

**Table 1:** Primers involved in this study

Name of primers	Sequence (5′ to 3′)	References
PCR primers		
rPLU1	TCAAAGATTAAGCCATGCAAGTGA	Snounou et al. [7]
rPLU5	CCTGTTGTTGCCTTAAACTCC	
PkF1140	GATTCATCTATTAAAAATTTGCTTC	Imwong et al. [9]
PkR1550	GAGTTCTAATCTCCGGAGAGAAAAGA	
LAMP primers		
FIP	GTTGTTGCCTTAAACTTCCTTGTGTTCTTGATTGTAAAGCTTCTTAGAGG	Lau et al. [10]
BIP	TGATGTCCTTAGATGAACTAGGCTTTGCAAGCAGCTAAAATCGT	
FLP	TAGACACACATCGTT	
BLP	GCACGCGTGCTACACT	
F3	CCATCTATTTCTTTTTTGCGTATG	
B3	CAGTGGAGGAAAAGTACGAA	

### SYBR green I LAMP

The SYBR green I LAMP assay was conducted using the primers targeted on the *18S rRNA* gene that has been described by Lau et al. [8]. The LAMP assay was performed in a 25-µL reaction mixture that consisted of 5.7 µL distilled water, 2.5 µL of 10X isothermal amplification buffer, 5.5 µL of MgSO_4_, 2.7 µL of dNTPs (New England Biolabs, Ipswich, Massachusetts, United States), 4 µL of betaine (Sigma-Aldrich, St. Louis, Missouri, United States), 40 pmol of FIP and BIP each, 10 pmol of FLP and BLP each, 5 pmol of F3 and B3 each, and 1 µL of *Bst* 2.0 WarmStart DNA polymerase (New England Biolabs, Ipswich, Massachusetts, United States). The template consisted of 4 µL of extracted DNA from blood spots. One µL of diluted SYBR green I (Sigma-Aldrich, St. Louis, Missouri, United States) was placed on the inner side of the tube. The closed-tube was then incubated at 65 °C in a Loopamp Real-Time Turbidimeter LA 500 (Eiken, Taiko-ku, Japan) for 30 min. At the end of the reaction, the tubes were cooled to room temperature and briefly spun to allow mixing of SYBR green I with the amplified products. The colour changes were visualized by the naked eyes. The kappa (k) statistics was applied to calculate the agreement between the results observed by the real-time turbidity meter and the colour changes.

### Analytical sensitivity and specificity

The analytical sensitivity of the SYBR green I LAMP assay was determined by amplifying the fragment via PCR using outer LAMP primers (F3 and B3) and cloned into pGEM-T vector (Promega, Madison, Wisconsin, United States). The recombinant plasmids were extracted using QIAprep Spin Miniprep kit (Qiagen, Hilden, Germany) and sent for sequencing to validate its identity. The detection limit of the SYBR green I LAMP assay was determined by calculating the copy number of the plasmid which was calculated based on the following formula: number of copies = (amount of plasmid (ng) × 6.022 × 10^23^)/(length of plasmid (bp) × 1 × 10^9^ × 650). A 10-fold serial dilution of the plasmid (10^6^ copies to one copy) was performed with sterile distilled water. One µL of each of the diluted DNA plasmids was used as the template. LAMP assay was repeated twice for each dilution.

Meanwhile, the specificity of SYBR green I LAMP assay was tested by using DNA template from 12 non-*P. knowlesi* parasites (4 *P. falciparum*, 4 *P. vivax*, 3 *Plasmodium malariae* and 1 *Plasmodium ovale*), 1 *Toxoplasma gondii*, 2 *Sarcocystis* spp., 2 *Brugia* spp. samples. The LAMP assay was repeated twice for each of the templates.

### Clinical sensitivity and specificity

The clinical sensitivity and specificity of nested PCR and the SYBR green I LAMP assay were evaluated using 71 *P. knowlesi* blood samples and 20 healthy donors with microscopy as the reference method. Sensitivity was calculated as (number of true positives)/(number of true positives + number of false negatives), and specificity was calculated as (number of true negatives)/(number of true negatives + number of false positives). In addition, 95% Confidence Intervals (95% CI) for both sensitivity and specificity were calculated using MEDCALC® software available at https://www.medcalc.org/calc/diagnostic_test.php.

## Results

The detection limit of the SYBR green I LAMP assay was 10 copies/µL. As each Plasmodium parasite will usually carry 5–10 copies of *18S rRNA* gene [9]. The detection limit of this assay was 1–2 parasites/µL. The detection limit of the SYBR green I LAMP assay was lower than nested PCR, which had a detection limit of 10–20 parasites/µL of *P. knowlesi*, well below the threshold of detection by microscopy with ~ 30 parasites/µL.

The analytical specificity of the SYBR green I LAMP assay was tested using DNA of non-human malaria species (*Plasmodium coatneyi*, *Plasmodium cynomolgi*, *Plasmodium fragile*, *Plasmodium brasilianum*, and *Plasmodium inui*), 12 non-*P. knowlesi* parasites (4 *P. falciparum*, 4 *P. vivax*, 3 *P. malariae* and 1 *P. ovale*), 1 *Toxoplasma gondii*, 2 *Sarcocystis* spp., 2 *Brugia* spp. samples. The results showed that none of these samples were amplified by SYBR green I LAMP assay. A representative gel image of LAMP assay was shown in Additional file 1.

The colour changes between positive and negative samples were blind tested by at least five observers (Additional file 2). LAMP assay detection using real-time turbidity meter and SYBR green I showed perfect agreement with kappa value of 0.98.

**Fig. 1:**
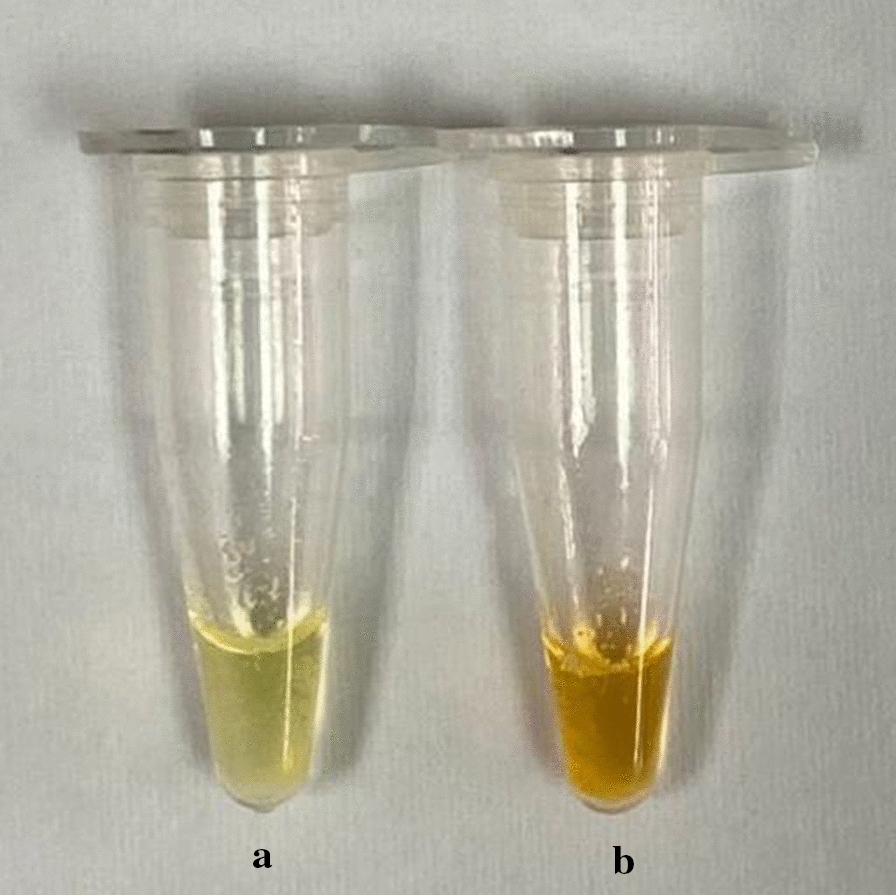
Detection of LAMP end products by colour changes. Positive LAMP reaction changed to green colour (**a**) while negative LAMP reaction remained orange (**b**)

A total of 71 microscopy positive samples were collected from Sarawak State Health Department. Twenty healthy donors were also collected from the same place. None of the healthy donors exhibited any symptoms of malaria. Out of 71 positive samples, 47 samples show positive by nested PCR while 69 samples show positive by SYBR green I LAMP assay (Additional file 3). Positive and negative reactions were indicated as green and orange, respectively (Fig. 1). Two samples were detected as negative by both nested PCR and SYBR green I LAMP assay. We observed that sample with higher parasitaemia were positive by both nested PCR and LAMP. However, 22 samples with low parasitaemia (< 0.34%) were not amplified by nested PCR. Both samples tested negative by LAMP and nested PCR had relatively low parasitaemia (0.3%). To further confirm the identity of the *P. knowlesi* samples (negative for nested PCR but positive for both microscopy and SYBR green I LAMP assay), these samples were subjected to a simple PCR amplification (using F3 and B3 primers), the products were cloned into pGEM-T vector (Promega, Madison, USA) and sent for sequencing. Results show that these samples were 100% similar to *P. knowlesi*.

For clinical sensitivity and specificity test, the SYBR green I LAMP assay did not detect any of the negative DNA samples. It was 97.1% sensitive and 100% specific (Table 2).

**Table 2:** Overall sensitivity and specificity of the nested PCR and LAMP assay

Methods	*P. knowlesi*	Healthy donor	Overall Sensitivity (%) (95% CI)	Overall Specificity (%) (95% CI)
Microscopy				
Positive	71	0		
Negative	0	20		
Nested PCR				
Positive	47	0	66.2 (54–77)	100 (83.2–100)
Negative	24	20		
LAMP				
Positive	69	0	97.1 (90.2–99.7)	100 (83.2–100)
Negative	2	20		

The concentration of SYBR green I dye was optimized by preparing different concentrations of SYBR green I (1000X, 100X, 50X, 20X and 10X) were prepared from stock (10,000X). One µL of diluted SYBR green I was added to the tube with completed reaction. A positive reaction results in a green colour as the dye intercalates in the nucleic acid while the negative reaction remains orange indicating no amplification has taken place. Following the experiment, 20X and 10X concentration of SYBR green I showed positive results. In order to save reagent and cost, 10X concentration of SYBR green I is recommended to be used.

## Discussion

Compared to microscopy, nested PCR was 66.2% sensitive and 100% specific. The SYBR green I LAMP assay exhibited higher sensitivity (97.1%) than nested PCR (Table 2). Also, among the 71 collected *P. knowlesi* samples, the SYBR green I LAMP assay managed to detect 69 *P. knowlesi* samples while nested PCR only detected 47 *P. knowlesi* samples. The findings presented in this study show the SYBR green I LAMP assay is more sensitive than nested PCR for detection of *P. knowlesi*. Additionally, the detection limit of *P. knowlesi* SYBR green I LAMP assay was 10 copies which was lower than nested PCR, which had a detection limit of 100 copies. The nested PCR in this study may be hampered by suboptimal reaction conditions, including utilization of poor quality *Taq* polymerase.

The F3 and B3 primers used here was 100% specific for *P. knowlesi*. The primer sequences do not share sequence identity with all other human malaria species including closely related *P. vivax* [10]. The analytical specificity of LAMP showed no cross-reactivity with non-*P. knowlesi* parasites.

The diagnostic accuracy of LAMP assay was found to be different when using different target genes for *P. knowlesi*. By using beta tubulin as target gene, Iseki et al. managed to detect down to 100 copies/µL DNA template [11]. While Lau et al. managed to detect down to 10 copies/µL of DNA template by using AMA-1 as the target gene. While using mitochondrial as the target gene, Britton et al. managed to detect 1.4 parasites/µL as lowest detection limit [12]. The present study managed to detect down to 10 copies/µL DNA template by using *18S rRNA* as target gene.

SYBR Green I was used as the fluorescence dye in this study due to its visualization via observation colour changes is significant in comparison to other green fluorescence dye such as calcein-manganese II chloride (MnCl_2_) dye. Moreover, calcein-MnCl2 methodology is less sensitive [13]. Results from the present study indicate that 10X dilution of SYBR was optimal for this assay.

SYBR green dye is not recommended to be added into the reaction mixture before the start of reaction as it inhibits the reaction [14]. Therefore, SYBR green I is suggested to be added into reaction tube upon the completion of the LAMP amplification. Green and orange colour end products indicate positive and negative result, respectively. The method from this study possess an advantage over other published closed-tube LAMP assay. Karthik et al. developed a closed-tube LAMP assay based on agar dye capsule [15]. However, this method was tedious as it needed a high temperature to melt the molten agar and required more materials to perform the LAMP assay. The closed tube SYBR green I LAMP assay developed in the present study was simple and did not require a lot of materials and time.

In 2011, Tao et al. reported an established visualized closed-tube LAMP method for field detection of *P. vivax* [16]. The method was performed by incorporation of a microcrystalline wax-dye capsule containing the SYBR Green I dye to a normal LAMP reaction prior to the initiation of the reaction. This microcrystalline wax-dye capsule methodology is laborious, time consuming and requires a PCR machine for wax melting if compared to SYBR green I LAMP assay in the present study. Thus, the method in this study is highly potential to be developed for the use in resource-limited areas, specifically in malaria endemic regions.

In the current format of SYBR green I LAMP assay, carry-over contamination can be eliminated. It was because the cap of the tube was not necessary to open since the dye was added into inner cap of the tube prior to the start of amplification. Following the amplification, the tube was briefly spun to allow mixing of SYBR green I with the amplified products. Positive and negative reaction indicate green and orange colour, respectively that can be visualized by naked eyes. The SYBR green I LAMP developed here was different from other studies, at which SYBR green I dye was added into the reaction tube upon completed amplification [17, 18]. The SYBR green I LAMP assay here also did not require the preparation of agar-dye capsule or wax-dye capsule as in other studies. This will help to save both timing and cost reagents.

## Conclusions

Rapid, reliable, and species-specific diagnostic tools are indispensable for effective control of malaria. Light microscopy-based diagnosis to date is the gold standard method. However, it demands technical expertise and suffers low detection limits in field conditions. SYBR green I LAMP assay is a robust method for the detection of human *P. knowlesi*. Therefore, the SYBR green I LAMP assay has potential for use in resource-limited settings.

### Supplementary Information

**Additional file 1: Figure S1. **A representative gel image of LAMP products. Lane 1: Ladder (100 bp); Lane 2 to Lane 4: positive reaction of LAMP assay; Lane 5 to 7: negative LAMP reaction; Lane 8: negative control (distilled water).

**Additional file 2: Figure S2. **Colour changes of LAMP product by additional of SYBR green I into the LAMP reaction. Tube 1 and 3 are positive reactions. Tube 2 and 4 are negative reactions. (A): obviously positive; (B): less intense result.

**Additional file 3: Table S1.** Microscopy, PCR and LAMP results.

## Data Availability

The data generated or analysed during this study are either included in this published article.

## Abbreviations

LAMP: Loop-mediated isothermal amplification
DNA: Deoxyribonucleic acid
