# Correction: Validation of SYBR green I based closed‐tube loop‐mediated isothermal amplification (LAMP) assay for diagnosis of knowlesi malaria

**DOI:** 10.1186/s12936-023-04731-y

**Published:** 2023-10-18

**Authors:** Meng Yee Lai, Choo Huck Ooi, Yee Ling Lau

**Affiliations:** 1 Department of Parasitology, Faculty of Medicine, University of Malaya, 50603 Kuala Lumpur, Malaysia; 2 Sarawak State Health Department, Jalan Diplomatik, Off Jalan Bako, 93050 Kuching, Sarawak Malaysia

**Correction: Malaria Journal (2021) 20:166** **https://doi.org/10.1186/s12936-021-03707-0**

Following publication of the original article [1], it was brought to the journal's attention that the Funding declaration was incomplete. The declaration has since been corrected to the following: This study was supported by the Long Term Research Grant Scheme (LRGS/1/2018/UM/01/1/4) of the Ministry of Higher Education, Malaysia.

The authors thank you for reading this erratum and apologize for any inconvenience caused.
