# Supplementary material for: Validation of SYBR green I based closed‐tube loop‐mediated isothermal amplification (LAMP) assay for diagnosis of knowlesi malaria
Source: Malar J. 2021 Mar 25;20:166. doi: 10.1186/s12936-021-03707-0 (PMC7995794; doi:10.1186/s12936-021-03707-0)
Supplement: Supplementary file 1 — Additional file 1: Figure S1. A representative gel image of LAMP products. Lane 1: Ladder (100 bp); Lane 2 to Lane 4: positive reaction of LAMP assay; Lane 5 to 7: negative LAMP reaction; Lane 8: negative control (distilled water). [file 12936_2021_3707_MOESM1_ESM.docx]

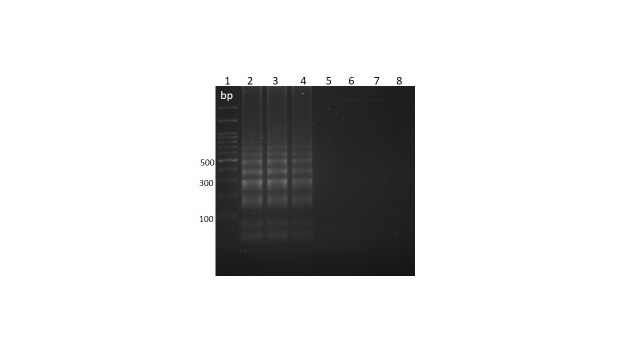


Figure S1. A representative gel image of LAMP products. Lane 1: Ladder (100 bp); Lane 2 to Lane 4: positive reaction of LAMP assay; Lane 5 to 7: negative LAMP reaction; Lane 8: negative control (distilled water).
