# Supplementary material for: Validation of SYBR green I based closed‐tube loop‐mediated isothermal amplification (LAMP) assay for diagnosis of knowlesi malaria
Source: Malar J. 2021 Mar 25;20:166. doi: 10.1186/s12936-021-03707-0 (PMC7995794; doi:10.1186/s12936-021-03707-0)
Supplement: Supplementary file 2 — Additional file 2: Figure S2. Colour changes of LAMP product by additional of SYBR green I into the LAMP reaction. Tube 1 and 3 are positive reactions. Tube 2 and 4 are negative reactions. (A): obviously positive; (B): less intense result. [file 12936_2021_3707_MOESM2_ESM.docx]

Figure S2. Colour changes of LAMP product by additional of SYBR green I into the LAMP reaction. Tube 1 and 3 are positive reactions. Tube 2 and 4 are negative reactions. (A): obviously positive; (B): less intense result.
