# Supplementary material for: Validation of SYBR green I based closed‐tube loop‐mediated isothermal amplification (LAMP) assay for diagnosis of knowlesi malaria
Source: Malar J. 2021 Mar 25;20:166. doi: 10.1186/s12936-021-03707-0 (PMC7995794; doi:10.1186/s12936-021-03707-0)
Supplement: Supplementary file 3 — Additional file 3: Table S1. Microscopy, PCR and LAMP results. [file 12936_2021_3707_MOESM3_ESM.docx]

**Additional file 3**

**Table S1: Microscopy, PCR and LAMP results.**

| Sample | Microscopy | Parasitemia (%) | PCR | LAMP |
| --- | --- | --- | --- | --- |
| BS0023 | Positive | 0.58 | Positive | Positive |
| BS1054 | Positive | 3.84 | Positive | Positive |
| BS0979 | Positive | 2.29 | Positive | Positive |
| BS0035 | Positive | 0.32 | Negative | Positive |
| BS0016 | Positive | 0.31 | Negative | Positive |
| BS0052 | Positive | 0.33 | Negative | Positive |
| BS0024 | Positive | 0.32 | Negative | Positive |
| BS0390 | Positive | 1.4 | Positive | Positive |
| BS0625 | Positive | 1.6 | Positive | Positive |
| BS0758 | Positive | 0.98 | Positive | Positive |
| BS0783 | Positive | 3.4 | Positive | Positive |
| BS0040 | Positive | 0.34 | Negative | Positive |
| BS0877 | Positive | 1.79 | Positive | Positive |
| BS0876 | Positive | 1.15 | Positive | Positive |
| BS0884 | Positive | 0.31 | Positive | Positive |
| BS0032 | Positive | 0.3 | Negative | Negative |
| BS0904 | Positive | 1.88 | Positive | Positive |
| BS0925 | Positive | 1.29 | Positive | Positive |
| BS0056 | Positive | 1.26 | Positive | Positive |
| BS0252 | Positive | 0.33 | Negative | Positive |
| BS0012 | Positive | 0.31 | Negative | Positive |
| BS0031 | Positive | 1.91 | Positive | Positive |
| BS0088 | Positive | 0.96 | Positive | Positive |
| BS0102 | Positive | 2 | Positive | Positive |
| BS0108 | Positive | 1.44 | Positive | Positive |
| BS0049 | Positive | 0.33 | Negative | Positive |
| BS0034 | Positive | 0.3 | Negative | Negative |
| BS0261 | Positive | 1.92 | Positive | Positive |
| BS0029 | Positive | 0.31 | Negative | Positive |
| BS0217 | Positive | 1.92 | Positive | Positive |
| BS0282 | Positive | 0.48 | Positive | Positive |
| BS0288 | Positive | 2.14 | Positive | Positive |
| BS0410 | Positive | 2.03 | Positive | Positive |
| BS0070 | Positive | 1.4 | Positive | Positive |
| BS0633 | Positive | 2.95 | Positive | Positive |
| BS0022 | Positive | 0.79 | Positive | Positive |
| BS0472 | Positive | 1.93 | Positive | Positive |
| BS0117 | Positive | 0.32 | Negative | Positive |
| BS1022 | Positive | 1.79 | Positive | Positive |
| BS0784 | Positive | 1.6 | Positive | Positive |
| BS0647 | Positive | 1.32 | Positive | Positive |
| BS0724 | Positive | 1.19 | Positive | Positive |
| BS0763 | Positive | 1.99 | Positive | Positive |
| BS0700 | Positive | 1.59 | Positive | Positive |
| BS0525 | Positive | 0.42 | Positive | Positive |
| BS0882 | Positive | 1.26 | Positive | Positive |
| BS1028 | Positive | 1.67 | Positive | Positive |
| BS0089 | Positive | 0.32 | Negative | Positive |
| BS0438 | Positive | 3.81 | Positive | Positive |
| BS0935 | Positive | 0.84 | Positive | Positive |
| BS0098 | Positive | 0.31 | Negative | Positive |
| BS1132 | Positive | 1.56 | Positive | Positive |
| BS0964 | Positive | 1.98 | Positive | Positive |
| BS0189 | Positive | 1.96 | Positive | Positive |
| BS0116 | Positive | 0.32 | Negative | Positive |
| BS0038 | Positive | 0.31 | Negative | Positive |
| BS0298 | Positive | 2.31 | Positive | Positive |
| BS0407 | Positive | 0.32 | Negative | Positive |
| BS0127 | Positive | 0.34 | Negative | Positive |
| BS0316 | Positive | 1.3 | Positive | Positive |
| BS0868 | Positive | 0.32 | Negative | Positive |
| BS0402 | Positive | 2.05 | Positive | Positive |
| BS0902 | Positive | 0.32 | Negative | Positive |
| BS0529 | Positive | 3.46 | Positive | Positive |
| BS0146 | Positive | 0.33 | Negative | Positive |
| BS0688 | Positive | 1.29 | Positive | Positive |
| BS0151 | Positive | 0.33 | Negative | Positive |
| BS0684 | Positive | 3.8 | Positive | Positive |
| BS0234 | Positive | 0.32 | Negative | Positive |
| BS0229 | Positive | 0.34 | Negative | Positive |
| BS1128 | Positive | 2.3 | Positive | Positive |
